# Bark Beetle Attack History Does Not Influence the Induction of Terpene and Phenolic Defenses in Mature Norway Spruce (*Picea abies*) Trees by the Bark Beetle-Associated Fungus *Endoconidiophora polonica*

**DOI:** 10.3389/fpls.2022.892907

**Published:** 2022-05-06

**Authors:** Raimund Nagel, Almuth Hammerbacher, Grit Kunert, Michael A. Phillips, Jonathan Gershenzon, Axel Schmidt

**Affiliations:** Department of Biochemistry, Max Planck Institute for Chemical Ecology, Jena, Germany

**Keywords:** terpenes, stilbenes, flavonoids, flavan-3-ols, isoprenyl diphosphate synthase, defense priming

## Abstract

Terpenes and phenolics are important constitutive and inducible conifer defenses against bark beetles and their associated fungi. In this study, the inducible defenses of mature Norway spruce (*Picea abies*) trees with different histories of attack by the spruce bark beetle, *Ips typographus* were tested by inoculation with the *I. typographus*-associated fungus *Endoconidiophora polonica*. We compared trees that had been under previous attack with those under current attack and those that had no record of attack. After fungal inoculation, the concentrations of mono-, sesqui-, and diterpenes in bark increased 3- to 9-fold. For the phenolics, the flavan-3-ols, catechin, and gallocatechin, increased significantly by 2- and 5-fold, respectively, while other flavonoids and stilbenes did not. The magnitudes of these inductions were not influenced by prior bark beetle attack history for all the major compounds and compound classes measured. Before fungal inoculation, the total amounts of monoterpenes, diterpenes, and phenolics (constitutive defenses) were greater in trees that had been previously attacked compared to those under current attack, possibly a result of previous induction. The transcript levels of many genes involved in terpene formation (isoprenyl diphosphate synthases and terpene synthases) and phenolic formation (chalcone synthases) were significantly enhanced by fungal inoculation suggesting *de novo* biosynthesis. Similar inductions were found for the enzymatic activity of isoprenyl diphosphate synthases and the concentration of their prenyl diphosphate products after fungal inoculation. Quantification of defense hormones revealed a significant induction of the jasmonate pathway, but not the salicylic acid pathway after fungal inoculation. Our data highlight the coordinated induction of terpenes and phenolics in spruce upon infection by *E. polonica*, a fungal associate of the bark beetle *I. typographus*, but provide no evidence for the priming of these defense responses by prior beetle attack.

## Introduction

Conifers throughout the world have come under increasing attack from bark beetles in recent years as a result of global warming. Rising temperatures have promoted beetle reproduction while increasing stress on the trees themselves due to heat and drought (Biedermann et al., 2019). In central Europe, the dominant conifer Norway spruce (*Picea abies*) is attacked by the Eurasian spruce bark beetle, *Ips typographus*, which vectors various fungi that contribute to its successful attack. The ascomycete *Ceratocystis polonica*, recently renamed as *Endoconidiophora polonica* (De Beer et al., 2014), is considered the most virulent (Wermelinger, 2004). The partnership between beetles and their fungal symbionts is lethal for many hectares of spruce forest each year.

**FIGURE 1:**
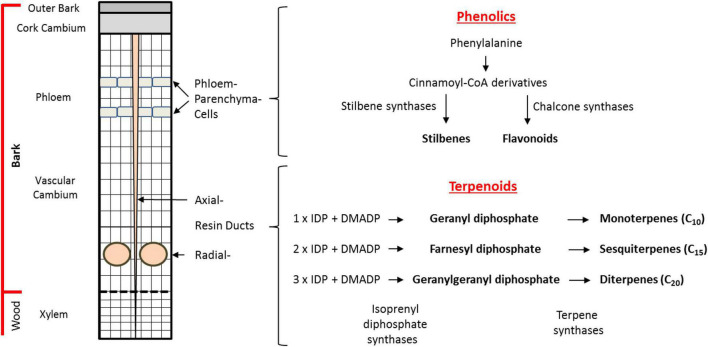
Biosynthesis and storage of defense compounds in Norway spruce bark. Depicted on the left is a schematic cross section of the cambial region of the trunk of a mature tree. Phenolic compounds are localized in bands of phloem parenchyma cells in the phloem. Terpenoid resin compounds are localized in radial resin ducts in the xylem and in axial resin channels that traverse both the xylem and phloem. An outline of the biosynthetic pathways to terpene and phenolic defense compounds is given on the right. The major phenolic compounds (stilbenes, flavonoids) are synthesized in the phloem parenchyma cells from phenylalanine with stilbene synthases (STSs) and chalcone synthases (CHSs) being the key branchpoint enzymes. The terpenoid resin compounds are synthesized in the epithelial cells lining the resin ducts from the C_5_ units isopentenyl diphosphate (IDP) and dimethylallyl diphosphate (DMADP). The isoprenyl diphosphate synthases catalyze the formation of the intermediates geranyl diphosphate (GDP), farnesyl diphosphate (FDP), and geranylgeranyl diphosphate (GGDP). These intermediates are converted into monoterpenes (C_10_), sesquiterpenes (C_15_), and diterpenes (C_20_), respectively, by the action of terpene synthases (TPSs).

Norway spruce possesses various modes of defense against beetle-fungal attack. Besides the physical barrier of the bark consisting of lignified and suberized cells, chemical barriers in the bark include the presence of terpene-based oleoresin accumulating in axial and radial resin ducts and phenolic compounds stored in phloem parenchyma cells (Figure 1). Although terpenes and phenolics are present constitutively in Norway spruce, their accumulation increases upon bark beetle attack or artificial inoculation with *E. polonica* (Viiri et al., 2001; Lieutier et al., 2003; Franceschi et al., 2005; Zhao et al., 2010; Hammerbacher et al., 2011, 2014; Wadke et al., 2016). In addition, application of the defense hormone methyl jasmonate results in significant accumulation of terpene and phenolic defenses (Martin et al., 2002; Erbilgin et al., 2006; Zeneli et al., 2006; Schmidt et al., 2011; Schiebe et al., 2012).

Spruce oleoresin terpenes include monoterpenes (C_10_), sesquiterpenes (C_15_), and diterpenes (C_20_). These compounds are synthesized from the C_5_ building blocks, isopentenyl diphosphate (IDP), and dimethylallyl diphosphate (DMADP), produced either by the cytosolic mevalonate pathway (MVA) or the plastidial methylerythritol phosphate (MEP) pathway. The C_5_ building blocks are assembled by isoprenyl diphosphate synthases (IDS), members of an enzyme class known as prenyltransferases, which catalyze the sequential head-to-tail condensation of 1–3 IDP molecules with DMADP producing geranyl diphosphate (GDP, C_10_), farnesyl diphosphate (FDP, C_15_), and geranylgeranyl diphosphate (GGDP, C_20_) (Figure 1; Wang and Ohnuma, 2000; Liang et al., 2002; Schmidt and Gershenzon, 2007, 2008; Schmidt et al., 2010, 2011; Nagel et al., 2019). GDP, FDP, and GGDP are substrates for terpene synthases (TPS), which produce an array of terpene skeletons that can be further modified, for example, *via* the formation of copalyl diphosphate (CDP) and by cytochrome P450s generating the diverse chemical composition of spruce oleoresin (Keeling and Bohlmann, 2006; Bohlmann and Keeling, 2008; Chen et al., 2011; Hall et al., 2013). In order to gain a deeper understanding how terpene biosynthesis is regulated under different conditions, such as after bark beetle attack, it is useful to study the expression of genes encoding IDS and TPS, measure enzyme activity and determine the *in vivo* concentrations of the key intermediates GDP, FDP, and GGDP (Nagel et al., 2012, 2014).

In addition to terpenoids, phenolics also constitute an important group of defensive metabolites in spruce. The major phenolic compounds accumulating in Norway spruce are stilbenes and flavonoids (Figure 1). These compounds are both biosynthesized by condensation of *para*-coumaroyl coenzyme A (CoA), formed from phenylalanine *via* the phenylpropanoid pathway, with malonyl CoA units formed from the polyketide pathway. These condensation reactions are catalyzed by stilbene synthase (STS) and chalcone synthase (CHS) enzymes to form a related tetraketide. However, STS enzymes cyclize the tetraketide to the tetrahydroxystilbene, resveratrol, which is further modified *via* glycosylation, oxidation, and methylation to the compounds astringin and isorhapontin, the predominant stilbenes in the bark of *P. abies* (Hammerbacher et al., 2011). Meanwhile, cyclization of the tetraketide by CHS yields the flavanone naringenin (Cheynier et al., 2013) which is further modified *via* two oxidation reactions to form the dihydroflavonol taxifolin that accumulates in spruce bark and needles. Further modification of taxifolin *via* two consecutive reductions yields the flavan-3-ol catechin, the precursor for proanthocyanidins (condensed tannins) (Hammerbacher et al., 2014). However, our knowledge of how phenolic biosynthesis is regulated in spruce after insect or pathogen attack is still limited.

As only mature spruce trees with a minimum bark thickness are attacked by bark beetles, large trees growing under natural conditions represent the most relevant study system in which to investigate defensive responses to beetles and their associated fungi. However, most previous experiments on spruce defenses have been performed on young saplings after application of methyl jasmonate (MJ) to induce defense responses (Lieutier et al., 2003; Erbilgin et al., 2006; Hammerbacher et al., 2011, 2013, 2014). Among studies on mature trees (Viiri et al., 2001; Zeneli et al., 2006; Zhao et al., 2010; Schiebe et al., 2012), few have tested the direct inoculation of a bark beetle-associated fungus, and no study has comprehensively quantified oleoresin terpenes and phenolic compounds in parallel while linking compound accumulation to levels of defense hormones and biosynthetic genes, enzymes and intermediates.

Previous research on plant-insect interactions in many systems has indicated that initial herbivore or pathogen attack may lead to greater resistance to subsequent attack by inducing the accumulation of defense compounds or priming plants to respond more vigorously on subsequent attack (Aranega-Bou et al., 2014). In Norway spruce, pre-treatment with the defense hormone MJ or a sublethal dose of bark beetle-associated fungus can lead to enhanced defensive responses to the subsequent invasion of bark beetles (Erbilgin et al., 2006; Zhao et al., 2011; Mageroy et al., 2020). However, it is still unclear if trees from previously attacked stands are also more resistant to future attacks and whether this is due to priming of defenses because of greater accumulation of oleoresin terpenes and phenolics or priming of defense responses. Thus we investigated defense responses in Norway spruce trees with different histories of bark beetle attack: (a) trees from a stand that were the sole survivors of a previous attack, (b) trees under actual, current attack, and (c) trees without any previous or current attack. All trees studied showed a similar magnitude of terpene and phenolic induction after fungal inoculation regardless of their attack history. Induction was accompanied by an up-regulation of genes, enzymes, and metabolic intermediates involved in their biosynthesis.

## Materials and Methods

### Chemicals

Isopentenyl diphosphate (IDP), dimethylallyl diphosphate (DMADP), geranyl diphosphate (GDP), farnesyl diphosphate (FDP), geranylgeranyl diphosphate (GGDP), 1,9-decadiene, ammonium bicarbonate, chlorogenic acid, formic acid, tert-butyl methyl ether (TBME), methanol and acetonitrile (LC-MS grade) were purchased from Sigma-Aldrich (St. Louis, MO, United States). Trimethylsulfonium hydroxide (TMSH) was ordered from Macherey-Nagel (Düren, Germany).

### Biological Material, Fungal Inoculation, and Sampling

Bark from 15 mature (approximately 80-year-old, diameter of 70 cm at the height of 1.30 m) spruce trees (*P. abies*), which were sole survivors of a massive bark beetle attack in 2008, was collected in the Bohemian Forest National Park near Plöckenstein, Austria (48.46°N–13.50°E) in June 2012. These trees were still surrounded by dead trees, were not subject to any bark beetle attacks between 2008 and 2012 (personal communication from the local forester), and had no apparent above-ground signs or symptoms of biotic and physical injury. They were designated as trees with a history of **p**revious **a**ttack (PA). At the same time, 19 trees of approximately the same age were sampled in the Kalkalpen National Park close to Bodinggraben, Austria (47.47°N–14.24°E) that were being attacked by bark beetles at the time of sampling. These trees are designated as trees under **c**urrent **a**ttack (CA). In the same stand, six trees not affected by the current outbreak were sampled. These trees had never been recorded to be attacked by bark beetles (personal communication from the local forester), and also had no apparent above-ground signs or symptoms of biotic and physical injury. They were designated as trees with **n**o **a**ttack (NA). All trees selected for the experiment were found in stands dominated by Norway spruce, typical for a mountain habitat in Central Europe at an elevation of 1300 m (4265 ft) above sea level, and did not show any obvious phenotypical differences.

At the onset of the experiment, bark samples including outer bark, cork cambium, phloem, and vascular cambium (for details see Figure 1) with a size of 1.5 × 10 × 0.7 cm (W × H × D) were cut from the stem with a knife at a height of approximately 1.50 m above the soil. At the same time, an 8-mm bark plug was removed on the opposite side of the sampled stem with a cork borer and inoculated with *E. polonica* culture 1993-208/115 using the method of Hammerbacher et al. (2011). Bark samples were then taken from these inoculated trees 14 d afterward. All sampled material was frozen immediately after harvest in dry ice and stored at −80°C. The experimental approach is illustrated in Supplementary Figure 1.

### Resin Terpene Quantification

The protocol was adapted from Lewinsohn et al. (1993). In brief, 100 mg of frozen ground plant material was extracted with 1 mL TBME containing 50 μg mL^–1^ 1,9-decadiene and 47.3 μg mL^–1^ dichlorodehydroabietic acid as internal standards under continuous shaking for 24 h. The extract was removed, washed with 0.3 mL of 0.1 M (NH_4_)_2_CO_3_, pH 8.0, and dried by using a Pasteur pipette filled with 100 mg of Na_2_SO_4_. The Na_2_SO_4_ column was further washed with 0.5 mL of TBME. To 0.4 mL of extract, 50 μL of *N*-trimethylsulfonium hydroxide in MeOH was added for methylation of diterpenoid resin acids, while the rest of the extract was used for mono- and sesquiterpene analysis. Both extracts were subsequently analyzed by GC-MS and GC-FID. An Agilent 6890 series coupled to either an Agilent 5973 mass spectrometer or and FID detector were used. The column for both instruments was an Phenomenex ZB-5MSi (30 m, 0.25 mm, 0.25 μm), and the injector temperature was set at 280°C in split less mode with injections of 1 μL. Starting temperature for mono- and sesquiterpene analysis was 40°C, held for 4 min and increased by 5°C min^–1^ to 180°C, followed by a bake out at 280°C. Starting temperature for diterpene analysis was 140°C, held for 4 min and then increased by 5°C min^–1^ to 250°C followed by a bake out at 280°C. The extracted plant material was dried after removal of the liquid phase and the dry weight determined, with the fresh weight generally being 1.9–2.2 times the dry weight.

### Extraction and Quantification of Phenolic Compounds From Spruce

For extraction of phenolic compounds, Norway spruce tissue was ground to a fine powder in liquid nitrogen and lyophilized at 0.34 mbar pressure using an Alpha 1–4 LD plus freeze dryer (Martin Christ GmbH, Osterode, Germany). Approximately 20 mg dried tissue was extracted with 1.2 mL analytical grade methanol containing 80 μg mL^–1^ chlorogenic acid as internal standard for 4 h at 4°C. The extract was centrifuged at 13,000 × *g* and the supernatant was recovered. Insoluble material was re-extracted with 1 mL methanol for 16 h. Supernatants were combined and evaporated to dryness under a stream of nitrogen. Dried samples were re-dissolved in 1.2 mL methanol and diluted 20 times (v/v) with water.

Chromatography was performed on an Agilent 1200 HPLC system (Agilent). Separation was achieved on a XDB C18 column (1.8 μm, 50 mm × 4.6 mm; Agilent Technologies, Waldbrunn, Germany). Formic acid (0.05%) in water and acetonitrile were employed as mobile phases A and B, respectively. The elution profile was: 0–1 min, 100% A; 1–7 min, 0–65% B in A; 7–8 min 65–100% B in A; 8–9 min 100% B, and 9–10 min 100% A. The total mobile phase flow rate was 1.1 mL min^–1^ and the column temperature was maintained at 25°C.

An API 3200 tandem mass spectrometer (AB Sciex) equipped with a turbospray ion source was operated in the negative ionization mode. The instrument parameters were optimized by infusion experiments with pure standards of catechin, gallocatechin, proanthocyanidin B1, astringin, taxifolin, and quercetin glucoside. For isorhapontin, piceid, and taxifolin glucoside, partially purified plant extracts were used for optimization. The ion spray voltage was maintained at −4500 V. The turbo gas temperature was set at 700°C. Nebulizing gas was set at 70 psi, curtain gas at 25 psi, heating gas at 60 psi, and collision gas at 10 psi. For each target analyte, multiple reaction monitoring (MRM) was used to monitor parent ion-to-product ion formation: for catechin- *m/z* 299.9/109.1 [collision energy (CE)-34 V; declustering potential (DP) −30 V]; for gallocatechin- *m/z* 304.8/179 (CE −28 V; DP −39 V); for proanthocyanidin B1- *m/z* 576.9/289.1 (CE −30 V; DP −50 V); for astringin- *m/z* 404.9/243.1 (CE −38 V; DP −50 V); for isorhapontin- *m/z* 419/257.1 (CE −18 V; DP −25 V); for piceid- *m/z* 389/227 (CE −38 V; DP −50 V); for taxifolin glucoside- *m/z* 465/285 (CE −44 V; DP −55 V); for quercetin glucoside- *m/z* 463/301 (CE −40 V; DP −55 V); for taxifolin- *m/z* 303/125 (CE −28 V; DP −40 V). Both Q1 and Q3 quadrupoles were maintained at unit resolution. Analyst 1.5 software (AB Sciex) was used for data acquisition and processing. Linearity of compound detection for quantification was verified by external calibration curves for catechin, astringin, quercetin glucoside, and taxifolin. Flavan-3-ol concentrations were determined relative to the catechin calibration curve, stilbenes relative to the astringin calibration curve, flavonol glucosides relative to the quercetin glucoside calibration curve and taxifolin relative to the taxifolin calibration curve.

### Quantitative Real-Time PCR

RNA isolation and complementary DNA synthesis from a subset of trees (PA = 6, CA = 12, NA = 6) were carried out as described in Schmidt et al. (2011). Quantitative real-time PCR was done with Brilliant SYBR Green QPCR Master Mix (Stratagene) and 10 pmol forward and 10 pmol reverse primer. Primer sequences for *Pa*IDS1, *Pa*IDS4, and *Pa*IDS5 are given in Schmidt and Gershenzon (2007) and Schmidt et al. (2010). For all other genes the primer sequences are shown in Supplementary Table 1. PCR was performed using a Stratagene MX3000P thermocycler according to the instruction manual. Transcript abundance was normalized to the transcript abundance of ubiquitin as described in Schmidt et al. (2010).

### Protein Extraction and Quantification of Isoprenyl Diphosphate Synthase Enzyme Activity

Total protein extracts from bark tissue and protein quantification were done as described in Nagel et al. (2012). Enzyme assays were carried out using 10 μg of total protein in 200 μL of 25 mM 3-(*N*-morpholino)-2-hydroxypropanesulfonic acid (MOPSO) buffer at pH 7.2 with 10% (v/v) glycerol, 10 mM MgCl_2_ and 50 μM IDP and DMADP each (Sigma-Aldrich, St. Louis, MO, United States) for 2 h at 30°C. Quantification was done as described under *in vivo prenyl diphosphate quantification* below but with an injection volume of 1 μL and without MRMs for the internal standards GSDP and FSDP.

### *In vivo* Prenyl Diphosphate Quantification

A 1 g portion of plant material was extracted three times with 5 mL of methanol:water (7:3, v/v) containing 0.3 μg each of geranyl S-thiolodiphosphate (GSDP) and farnesyl S-thiolodiphosphate (FSDP) each (Echelon Biosciences Incorporated, Salt Lake City, UT, United States). Extracts were combined and purified using 150 mg (6 mL) Chromabond HR-XA columns (Macherey-Nagel) that had been conditioned with 5 mL methanol and 5 mL water. After application of the extract, the column was washed with 4 mL water followed by 5 mL methanol. Prenyl diphosphates were eluted with 3 mL 1 M ammonium formate in methanol, evaporated under a stream of nitrogen to dryness and dissolved in 300 μL water:methanol (1:1). Quantification was done using an Agilent 1260 HPLC system (Agilent Technologies, Waldbrunn, Germany) coupled to an API 5000 triple-quadrupole mass spectrometer (AB Sciex Instruments). For separation, a Zorbax Extended C-18 column (1.8 μm, 50 mm × 4.6 mm; Agilent Technologies, Waldbrunn, Germany) was used. The mobile phase consisted of 5 mM ammonium bicarbonate in water as solvent A and acetonitrile as solvent B, with the flow rate set at 1.2 mL min^–1^ and the column temperature kept at 20°C. Separation was achieved by using a gradient starting at 5% B, increasing to 70% B in 5 min and 100% B in 1 min (0.5-min hold), followed by a re-equilbration to 0% B for 1.5 min (1-min hold) before the next injection. The injection volume for samples and standards was 2 μl; autosampler temperature was 4°C. The mass spectrometer was used in the negative electrospray ionization (EI) mode. Optimal settings were determined using standards, except for CDP for which no commercial standard is available; instead the settings for GGDP were used. Levels of ion source gases 1 and 2 were set at 60 and 70 psi, respectively, with a temperature of 700°C. Curtain gas was set at 30 psi and collision gas was set at 7 psi, with all gases being nitrogen. Ion spray voltage was maintained at −4200 V. For each target analyte, MRM was used to monitor parent ion-to-product ion formation (DP, declustering potential; EP, entrance potential; CE, collision energy; CXP, collision cell exit potential): for GDP- *m/z* 312.9/79 (DP −40, EP −6, CE −38, CXP 0), for FDP- *m/z* 380.9/79 (DP −40, EP −3, CE −42, CXP 0), for GGDP and CDP- *m/z* 449/79 (DP −45, EP −10, CE −50, CXP 0), for GSDP- *m/z* 329/79 (DP −45, EP −6, CE −20, CXP −13) and 329/159 (DP −45, EP −6, CE −20, CXP −19) and for FSDP- *m/z* 379/79 (DP −75, EP −6, CE −68, CXP −15) and 379/159 (DP −75, EP −6, CE −24, CXP −17). Data analysis was performed using Analyst Software 1.6 Build 3773 (AB Sciex).

### Identification of Copalyl Diphosphate as a Product When Total Protein Extract Was Assayed With Geranylgeranyl Diphosphate

Protein isolation from spruce bark was done as described in Nagel et al. (2012). An enzyme assay was performed in 4 mL of 25 mM MOPSO buffer, pH 7.2, with 10% (v/v) glycerol, 10 mM MgCl_2_ with 60 μg GGDP and incubated for 10 h at 30°C. Then 1 mL of 5 N HCl was added to cleave the diphosphate, and the assay was extracted three times with 2 mL pentane each. For GC-MS analysis the combined phases were evaporated to 200 μL.

An Agilent 6890 series GC with an Agilent 5973 mass spectrometer and a Zebron ZB-5MS column (30 m × 0.25 m × 0.25 μm) (Phenomenex, Aschaffenburg, Germany) were used for detection. Two μL of sample were injected in splitless mode with an injector temperature of 250°C and a flow rate of 1 mL min^–1^ helium. Oven temperature was initially 40°C, then raised by 7°C min^–1^ to 280°C and held there for 5 min. ChemStation G1701 was used for data analysis.

To identify CDP and determine its stereochemistry, standards were obtained from *Oryza sativa* copalyl diphosphate synthase 4 (*Os*4; syn-CDP) (Xu et al., 2004), *Arabidopsis thaliana* copalyl diphosphate synthase (*At*CPS; ent-CDP) (Prisic et al., 2004) and *Abies grandis* abietadiene synthase D621A mutant (*Ag*AS:D621A; normal-CDP) (Prisic et al., 2004). These genes were expressed as described in Wu et al. (2012). Bacterial pellets were resuspended in 3 mL of buffer containing 20 mM MOPSO, pH 7.0, 10% (v/v) glycerol, and 10 mM MgCl_2_ and sonicated using a Sonopuls HD 2070 (Bandelin, Berlin, Germany) for 4 min, cycle 2, power 60%. The lysate was centrifuged at 14,000 × *g* for 30 min. A 200 μL portion of the supernatant was used for enzyme assays carried out in 1 mL of 25 mM MOPSO buffer, pH 7.2, with 10% (v/v) glycerol, 10 mM MgCl_2_ and 20 μg GGDP as substrate, and incubated for 10 h at 30°C.

The enzyme assays were either injected directly into an LC-MS/MS as described under Protein extraction and quantification of isoprenyl diphosphate synthase enzyme activity or 200 μL 5 N HCl were added to cleave the diphosphate. The assay was extracted three times with 1 mL pentane each. The pentane phase was combined and evaporated to 100 μL for GC-MS analysis as described above.

An all *cis*-GGDP standard was obtained from *Solanum lycopersicum cis*-prenyltransferase 2 *Sl*CPT2 expressed and purified as described in Schilmiller et al. (2009) and Akhtar et al. (2013).

### Phytohormone Analysis

A subset of trees (PA = 6, CA = 12, NA = 6) was used to analyze phytohormone levels in order to obtain a better understanding of the signaling pathways involved in the defense response to *E. polonica* inoculation. The phytohormone analysis was based on the procedure described by Schmidt et al. (2011). Approximately 0.15 g of ground bark was homogenized in 1 mL methanol spiked with 40 ng of [^2^H_2_]JA, 40 ng [^2^H_4_]SA, 40 ng [^2^H_6_]ABA, and 8 ng of JA-[^13^C_6_]Ile by shaking for 60 min. Homogenates were centrifuged at 20,000 × *g* for 20 min at 4°C, the methanol phase collected, and the homogenate re-extracted with 1.0 mL methanol. The organic phases were combined and the samples evaporated to dryness in a vacuum concentrator at 30°C. The dry residue was reconstituted in 0.5 mL of 70% (v/v) methanol/water and analyzed by LC-MS/MS. Chromatography was performed on an Agilent 1200 HPLC system (Agilent). Separation was achieved on a XDB C18 column (1.8 μm, 50 mm × 4.6 mm; Agilent Technologies, Waldbrunn, Germany). The mobile phase, comprised of solvent A (0.05% formic acid) and solvent B (acetonitrile), was used in a gradient of 0–0.5 min, 5% B; 0.5–9.5 min, 0–58% B; 9.5–9.52 min, 58–100% B; 9.52–11 min, 100% B; 11–11.1 min, 5% B, and 11.1–14 min, 5% B with a flow rate of 1.1 mL min^–1^. The column temperature was maintained at 25°C. An injection volume of 2 μL was used for all samples. An API 5000 tandem mass spectrometer (AB Sciex) equipped with a turbospray ion source was operated in negative ionization mode. The ion spray voltage was maintained at −4500 V. The turbo gas temperature was set at 700°C. Nebulizing gas was set at 60 psi, curtain gas at 25 psi, heating gas at 60 psi, and collision gas at 7 psi. For each analyte, MRM was used to monitor parent ion-to-product ion formation (DP, declustering potential; EP, entrance potential; CE, collision energy; CXP, collision cell exit potential): for [^2^H_4_]SA- *m/z* 141/97 (DP −35, EP −8, CE −22, CXP 0); for SA- *m/z* 137/93 (DP −35, EP −8, CE −22, CXP 0); for [^2^H_2_]JA- *m/z* 213/59 (DP −35, EP −9, CE −24, CXP 0); for JA- *m/z* 209/59 (DP −35, EP −9, CE −24, CXP 0); for [^2^H_6_]ABA- *m/z* 269/159 (DP −35, EP −12, CE −22, CXP −2); for ABA- *m/z* 263/153 (DP −35, EP −12, CE −22, CXP −2); for JA-[^13^C_6_]Ile- *m/z* 328/136 (DP −50, EP −4, CE −30, CXP −4); for JA-Ile- *m/z* 322/130 (DP −50, EP −4, CE −30, CXP −4); for OPDA- *m/z* 291/165 (DP −45, EP −12, CE −24, CXP −2); for IAA- *m/z* 174/130 (DP −25, EP −9, CE −14, CXP −2); for hydroxy-JA-Ile- *m/z* 338/130 (DP −50, EP −4, CE −30, CXP −4); for hydroxy-JA- *m/z* 225/59 (DP −35, EP −9, CE −24, CXP 0) and for carboxy-JA-Ile- *m/z* 352/130 (DP −50, EP −4, CE −30, CXP −4). Data analysis was performed using Analyst Software 1.6 Build 3773 (AB Sciex). JA, JA-Ile, ABA, and SA were quantified according to the labeled standards, while hydroxy-JA and OPDA were quantified using [^2^H_2_]JA with response factors of 1.0 and 0.5, respectively; IAA was quantified using [^2^H_6_]ABA and a response factor of 3.4, and hydroxy-JA-Ile and carboxy-JA-Ile were quantified using JA-[^13^C_6_]Ile and response factors of 1.

### Statistical Analysis

Isoprenyl diphosphate synthases enzyme activity, prenyl-diphospate abundances and relative expression of biosynthetic genes were analyzed using mixed effects models lme of the nlme library (Pinheiro et al., 2018) with bark beetle attack history and fungal infection (before and after fungal inoculation) as fixed effects and tree individuals as a random intercept. To account for the variance heterogeneity of the residuals, the varIdent variance structure was applied if necessary. Whether the different variance of bark beetle attack, fungal infection, or the combination of both factors should be incorporated into the model, was determined by comparing models with different variance structures with a likelihood ratio test and choosing the model with the smallest AIC. If necessary, data were transformed in order to achieve normality of residuals. The influences of the fixed effects were obtained by removing fixed effects one after another and comparing the simpler with the more complex model with a likelihood ratio test (Zuur et al., 2009). Differences between factor levels were determined by factor level reduction (Crawley, 2013). CDP abundance and CDP enzyme activity was only detectable after fungal infection. Whether the CDP abundance and enzyme activity differed between trees with different bark beetle attack histories was analyzed using a one-way anova followed by the Tukey HSD test. If necessary to achieve variance homogeneity and normality of residuals, data were log-transformed.

The influence of the bark beetle attack history on initial terpene concentrations was analyzed with one way analysis of variance (anova) or if variance homogeneity or normality of residuals (inspected graphically) were violated with the non-parametric Kruskal–Wallis rank sum test. In case of significant differences, the Dunn’s test (package “dunn.test,” Dinno, 2017) was applied to reveal group differences. Terpenes with concentrations below the detection limit in more than half of all samples in each bark beetle attack history group were defined as not present and were not analyzed. To analyze the changes in terpene, phenolic and phytohormone concentrations due to fungal inoculation, the respective concentrations before fungal inoculation were subtracted from the concentrations 14 days after fungal inoculation of the respective tree. Positive values indicated an increase and negative values indicated a decrease in concentration due to fungal inoculation. Values around 0 suggest no changes in concentration. Whether the changes in concentration differed between trees with different bark beetle attack histories was analyzed with one-way analyses of variance (anova) followed by a Tukey HSD test in case of significant differences. If either variance homogeneity or normality of residuals (inspected graphically) were violated, the non-parametric Kruskal–Wallis rank sum test was applied, followed by the Dunn’s test (package “dunn.test,” Dinno, 2017) in case of significant differences. Afterward, we analyzed whether the differences in terpene and phytohormone concentration were significantly different from 0 (indicating a significant increase or decrease of terpenes or phytohormones due to fungal infection). This was done for the concentrations of all trees, regardless of their bark beetle attack history, if there were non-significant differences among the tree groups in the previous analysis. If there were significant differences in concentration among the tree groups, this analysis was done separately for the three tree groups. A one-sample *t*-test was used in case data were normally distributed; otherwise the non-parametric Wilcoxon signed rank test with continuity correction was performed. When the three tree groups were analyzed separately, the Bonferroni correction was applied to control the family-wise error rate. For all analyzes the type I error due to multiple comparisons was controlled using the false discovery rate (FDR). Data were analyzed using R 3.4.4 (R Core Team, 2018).

## Results

### All Classes of Resin Terpenes in Bark Increased Several-Fold Upon Experimental Fungal Infection, but Increase Was Not Influenced by Bark Beetle Attack History

**FIGURE 2:**
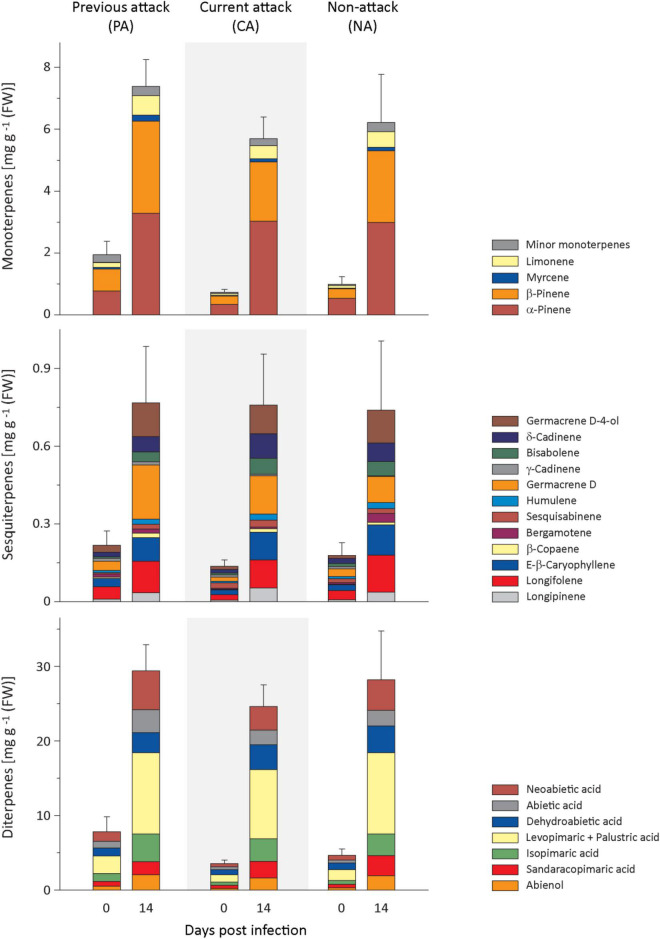
Induction of resin terpenes in spruce bark after *Endoconidiophora polonica* infection. The concentrations of the major resin monoterpenes **(upper panel)**, sesquiterpenes **(middle panel)**, and diterpenes **(lower panel)** were quantified by GC-FID analysis of bark tissue harvested before and 14 days after infection with *E. polonica*. Trees investigated: (1) were from a stand with a history of previous bark beetle attack, but no current attack (PA), (2) were under current attack, but had no previous history of attack (CA), or (3) had no previous nor current attack (NA). Data are means ± SE of measurements from 6 to 19 biological replicates; exact numbers are given in the Section “Materials and Methods.” Statistical values for individual compounds are given in Supplementary Tables 2, 3.

Resin terpenes were quantified to assess whether *E. polonica* inoculation of *P. abies* bark elicited a defense reaction. The total concentration of mono-, sesqui-, and diterpenes in the bark increased from approximately 0.65–3.3% of fresh weight (Figure 2). This increase was independent of the bark beetle attack history of the trees (Supplementary Table 2). However, total terpene concentrations prior to fungal infestation did differ significantly between trees with different bark beetle attack histories. Trees which survived a previous bark beetle attack (PA) had higher terpene concentrations than trees that were under current attack (CA), but neither PA nor CA trees were significantly different from non-attacked-trees (NA) (Supplementary Table 3).

Regarding monoterpenes, the concentrations of the four most abundant compounds, α-pinene, β-pinene, myrcene, and limonene, increased significantly by more than 4-fold 14 days after *E. polonica* infection, but the degree of increase was independent of bark beetle attack history (Figure 2 and Supplementary Table 2). The concentration before inoculation was significantly higher in PA trees than in CA trees for β-pinene, myrcene and limonene (Figure 2 and Supplementary Table 3). For minor terpenes, nearly all concentrations also increased significantly after *E. polonica* infection. Only α-phellandrene concentrations in CA trees and NA trees and *cis*-sabinene hydrate in NA trees did not change (Supplementary Figure 2 and Supplementary Table 2). Related to bark beetle history, the increase of concentrations between trees with different histories was only significantly different for the minor monoterpene *cis*-sabinene hydrate. Its increase was higher in PA trees than in CA trees (Supplementary Figure 2 and Supplementary Table 2). Similarly, the concentration of nearly all minor monoterpenes before inoculation was similar among trees with different histories, except that the concentrations of terpinolene and bornyl acetate were higher in PA trees than CA trees (Supplementary Figure 2 and Supplementary Table 3).

Out of the 12 sesquiterpenes, the concentrations of all but two, including the most abundant five, longifolene, (*E*)-β-caryophyllene, germacrene D, δ-cadinene, and germacrene D-4-ol, increased significantly after infection between 3- and 7-fold for samples from all attack histories (Figure 2 and Supplementary Table 2). The only compound showing a significantly different response in trees with different attack histories was *trans-*α-bergamotene, which did not increase at all in PA trees but slightly in NA trees after induction with *E. polonica*. The concentrations of sesquiterpenes before fungal inoculation were almost similar among trees with different attack histories, except that *trans-*α-bergamotene was higher in PA than in CA trees, while the opposite was true for sesquisabinene (Figure 2 and Supplementary Table 3).

The diterpene levels of all trees were significantly higher after *E. polonica* infection, increasing between 4− and 9-fold, but these increases were independent of the attack history of the trees (Figure 2 and Supplementary Table 2). However, the concentrations of diterpenes before fungal inoculation were similar among trees with different attack histories, except that abienol and neoabietic acid were significantly higher in PA trees than in CA trees (Figure 2 and Supplementary Table 3).

### Total Phenolic Levels Increased Upon Fungal Infection, but There Were Differences Among Phenolic Groups and Previously Attacked Trees Had Higher Levels of Total Phenolics

**FIGURE 3:**
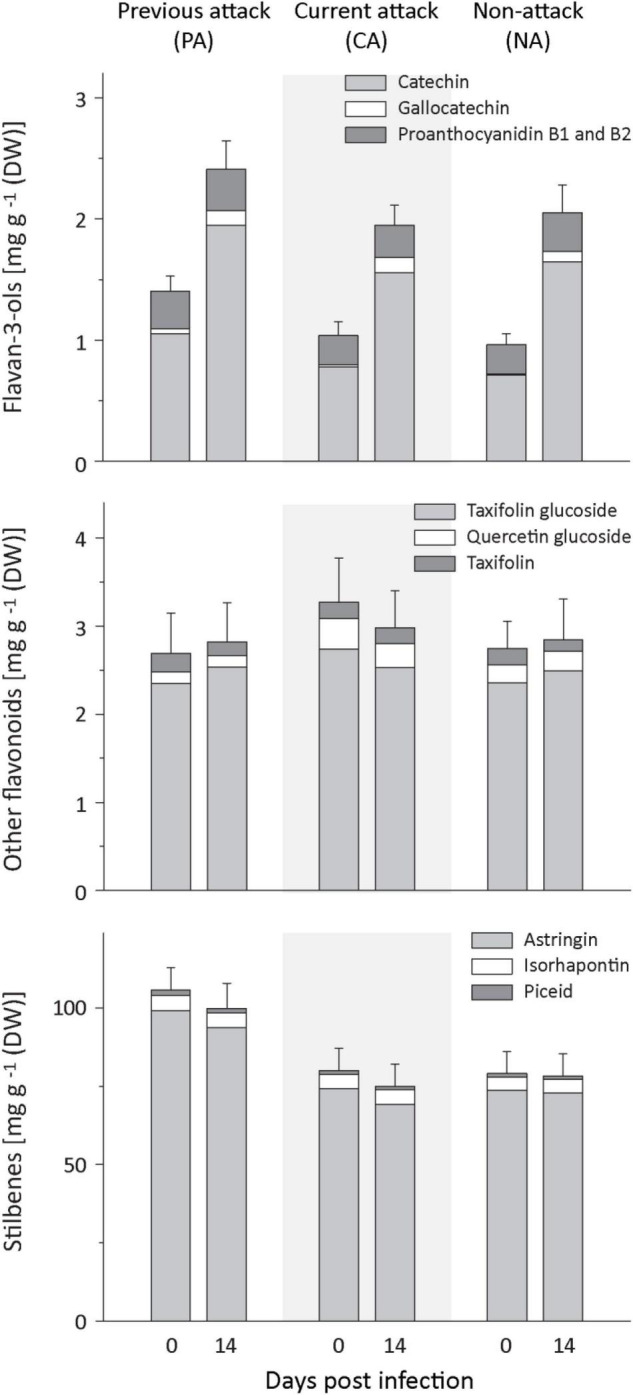
Induction of phenolic compounds in spruce bark after *Endoconidiophora polonica* infection. The concentration of flavan-3-ols **(upper panel)**, other flavonoids **(middle panel)**, and stilbenes **(lower panel)** were quantified by LC-MS/MS analysis from bark tissue harvested before and 14 days after infection with *E. polonica*. Trees investigated: (1) were from a stand with a history of previous bark beetle attack, but no current attack (PA), (2) were under current attack, but had no previous history of attack (CA), or (3) had no previous nor current attack (NA). Data are means ± SD of measurements from 6 to 19 biological replicates; exact numbers are given in the Section “Materials and Methods.” Statistical values for individual compounds are given in Supplementary Tables 4, 5.

Spruce contains several groups of phenolic compounds, including flavan-3-ols, other flavonoids and stilbenes. In response to fungal inoculation, the total amount of phenolics plus the amount of flavan-3-ols increased significantly, but there was no influence of bark beetle attack history on the level of increase (Figure 3 and Supplementary Table 4). Prior to fungal inoculation, PA trees had higher levels of total phenolics than CA or NA trees. The same pattern was found for the total stilbene level (Figure 3 and Supplementary Table 5). Total flavan-3-ols and the amounts of gallocatechin, but not the amounts of the flavan-3-ol dimers, were higher in PA than NA trees, but neither PA nor NA trees were significantly different from CA trees.

### Transcripts of Most Terpene and Phenolic Biosynthetic Genes Were Elevated in Response to Fungal Infection

To determine if terpene biosynthesis in *P. abies* bark was activated by *E. polonica* inoculation and how this was affected by bark beetle attack history, genes encoding three isoprenyl diphosphate synthases (*IDS*) and five terpene synthases (*TPS*) were selected for transcriptional studies. These genes encode proteins that catalyze the initial dedicated steps of resin formation (Martin et al., 2004; Schmidt and Gershenzon, 2007, 2008; Schmidt et al., 2010, 2011).

**FIGURE 4:**
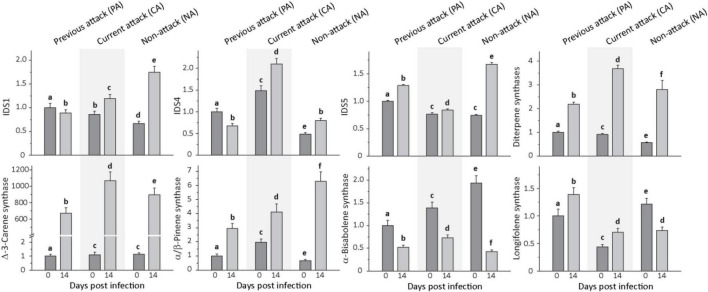
Relative expression of terpene biosynthetic genes before and after *Endoconidiophora polonica* infection. The expression of isoprenyl diphosphate synthase 1 (IDS1), IDS 4, IDS5, and various terpene synthases (diterpene synthases, δ-3-carene synthase, α/β-pinene synthase, α-bisabolene synthase, and longifolene synthase) were measured by quantitative real-time PCR analysis from bark tissue harvested before and 14 days after infection with *E. polonica*. Trees investigated: (1) were from a stand with a history of previous bark beetle attack, but no current attack (PA), (2) were under current attack, but had no previous history of attack (CA), or (3) had no previous nor current attack (NA). Different letters indicate significant differences. Data are means ± SD of measurements from 6 to 12 biological replicates; exact numbers are given in the Section “Materials and Methods.” Statistical values for individual compounds are given in Supplementary Table 6.

Transcript levels of all *P. abies IDS* measured were significantly altered by *E. polonica* infection in the investigated bark tissue (Figure 4 and Supplementary Table 6). Expression of *IDS1*, a bi-functional geranyl diphosphate and geranylgeranyl diphosphate synthase, *IDS4*, a farnesyl diphosphate synthase, and *IDS5*, a geranyl diphosphate synthase, increased significantly upon infection except for *IDS1* and *IDS4* in PA trees. When comparing the initial transcript level of *IDS* genes prior to fungal inoculation among trees with different attack histories, *IDS1* and *IDS5*, the genes involved in the biosynthesis of the most abundant resin constituents, the monoterpenes and diterpenes, had significantly greater transcript abundance in PA trees than in CA or NA trees. On the other hand, *IDS4*, which is involved in sesquiterpene biosynthesis, had significantly greater transcript levels in CA trees followed by PA trees and NA trees.

Expression of monoterpene and diterpene synthase genes was also significantly induced by fungal infection (Figure 4 and Supplementary Table 6). For the monoterpene synthase gene α/β-pinene synthase, transcript levels increased significantly by amounts ranging from 2- to 10-fold, depending on attack history, and δ-3-carene synthase transcripts increased by amounts ranging from 600- to over 900-fold, depending on attack history. Genes encoding the two diterpene synthase genes, isopimara-7,15-diene synthase and levopimaradiene/abietadiene synthase, were quantified together because of the high nucleotide sequence identity (∼ 95%) in the open reading frame. Transcripts increased significantly by amounts ranging from 2 to 5-fold, depending on attack history. Among the sesquiterpene synthase genes, expression of α-bisabolene synthase decreased significantly after *E. polonica* inoculation by about 2-fold in PA and CA trees, but more than 4-fold in NA trees. Expression of longifolene synthase increased in PA and CA trees by less than 2-fold, but decreased for NA trees by nearly 50% (Figure 4 and Supplementary Table 6).

**FIGURE 5:**
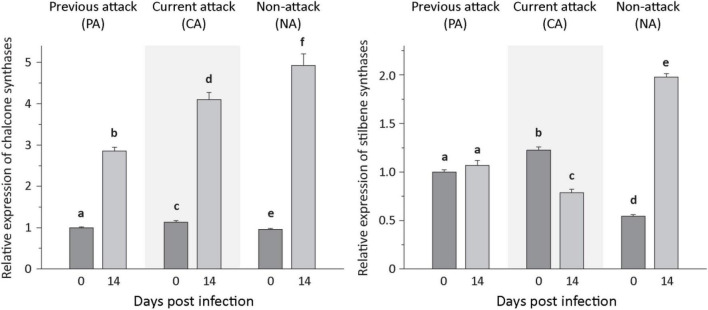
Relative expression of phenolic biosynthetic genes before and after *Endoconidiophora polonica* infection. The relative expression of chalcone synthases **(left panel)** and stilbene synthases **(right panel)** were measured by quantitative real-time PCR analysis from bark tissue harvested before and 14 days after infection with *E. polonica*. Trees investigated: (1) were from a stand with a history of previous bark beetle attack, but no current attack (PA), (2) were under current attack, but had no previous history of attack (CA), or (3) had no previous nor current attack (NA). Different letters indicate significant differences. Data are means ± SD of measurements from 6 to 12 biological replicates; exact numbers are given in the Section “Materials and Methods.” Statistical values for individual compounds are given in Supplementary Table 6.

For phenolic biosynthesis, we investigated genes encoding chalcone synthase, the first step in flavonoid formation, and genes encoding stilbene synthase, the first step in the biosynthesis of this type of phenolic compound. Expression of the chalcone synthases *CHS2*, *CHS6*, and *CHS8*, on the other hand, and the stilbene synthases *STS1* and *STS2*, on the other hand, were quantified together because of the high identity of the gene sequences at the nucleotide level. Expression of the three CHS genes increased significantly after inoculation with *E. polonica* by 2. 9−, 3. 8−, and 5.6-fold for PA, CA, and NA trees, respectively. In contrast, expression of the two *STS* genes was more variable; it was unaltered by fungal inoculation in PA trees, but decreased significantly by 1.5-fold in CA trees and increased significantly by 3.5-fold in NA trees (Figure 5 and Supplementary Table 6).

### Isoprenyl Diphosphate Synthase Activities and Levels of Isoprenyl Diphosphate Intermediates Increased Upon Fungal Infection

**FIGURE 6:**
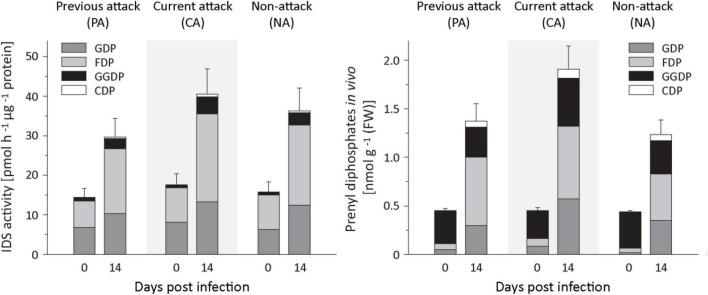
Quantification of IDS enzyme activity and prenyl diphosphate levels in spruce bark before and after *Endoconidiophora polonica* infection. The IDS enzyme activity of total protein extracts was assayed *in vitro* with IDP and DMADP as substrates (left panel). The abundance of the prenyl diphosphates GDP, FDP, GGDP, and CDP in spruce bark tissue *in vivo* was measured by LC-MS/MS. Trees investigated: (1) were from a stand with a history of previous bark beetle attack, but no current attack (PA), (2) were under current attack, but had no previous history of attack (CA), or (3) had no previous nor current attack (NA). Data are means ± SD of measurements from 6 to 12 biological replicates; exact numbers are given in the Section “Materials and Methods.” Statistical values for individual compounds are given in Supplementary Tables 7, 8.

To understand the regulation of terpenoid biosynthesis after fungal infection in Norway spruce bark, we followed up on the activation of *IDS* genes by looking for changes in IDS enzyme activity by assaying crude protein extracts *in vitro*. IDS activities showed significant increases after *E. polonica* infection with a 1. 8−, 1. 6−, and 2.2-fold increase in the rate of formation of GDP in PA, CA, and NA trees, respectively, a 2. 9−, 2. 5−, and 3.2-fold increase in FDP formation in these tree classes, respectively, and a 3. 3−, 8. 5−, and 22.6-fold increase of GGDP formation (Figure 6 and Supplementary Table 7). We also measured changes in the *in vivo* abundance of the prenyl diphosphate intermediates themselves by analysis of methanol-water extracts by LC-MS/MS. The *in vivo* levels of GDP and FDP increased significantly in response to infection with a 6−, 10. 9−, and 15.8-fold increase in GDP and 11. 5−, 15. 1−, and 15.8-fold increases in FDP in PA, CA, and NA trees, respectively, with a significant role of attack history. Increase in CA and NA trees was higher than in PA trees (Figure 6 and Supplementary Table 8). However, the *in vivo* level of GGDP did not change significantly after fungal infection.

**FIGURE 7:**
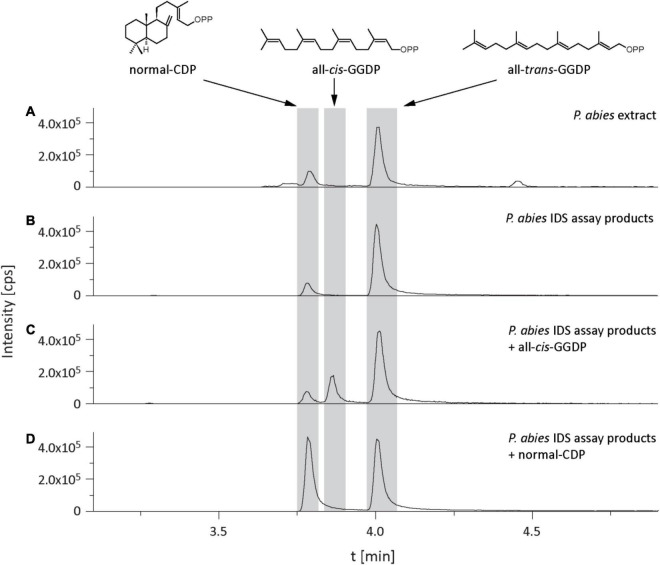
Identification of copalyl diphosphate (CDP) in *Picea abies* bark extracts and in IDS assays of total protein extracts with IDP and DMADP as substrates. LC-MS/MS chromatograms [m/z: 449/79] of extracts of **(A)** bark tissue, **(B)** IDS assay, **(C)** IDS assay plus all-*cis*-GGDP, **(D)** IDS assay plus *normal*-CDP.

In the isoprenyl diphosphate assays and analyses of plant extracts, we also identified another prenyl diphosphate in addition to GDP, FDP, and GGDP. This metabolite had the same mass and precursor-to-product ion formation as GGDP, but an earlier elution time (Figure 7). It was eventually identified as CDP, an intermediate in the formation of gibberellins and other diterpenes by comparison of retention time and mass spectrum with those of an authentic standard (Figure 7) as described previously (Nagel and Peters, 2017). Since separation of the stereoisomers of CDP *via* HPLC was not possible, this compound was analyzed by GC-MS after dephosphorylation and determined to be either the *normal*- or *ent*-isomer, which coelute (Wu et al., 2012) rather than the *syn*-isomer, which elutes separately (Supplementary Figure 3). CDP and CDP-synthesizing activity were only detected after *E. polonica* inoculation (Figure 6) and were significantly higher in CA trees than in PA and NA trees. CDP activity was lower in these PA than in CA trees, but both were similar to NA trees (Supplementary Tables 7, 8). The compound was presumably formed from GGDP in the total protein extract *via* a class II terpene synthase, and the changes observed indicate an elevated rate of diterpene biosynthesis.

### Jasmonates Increased Upon Fungal Infection, but Salicylic Acid, Indole-3-Acetic Acid, and Abscisic Acid Did Not

**FIGURE 8:**
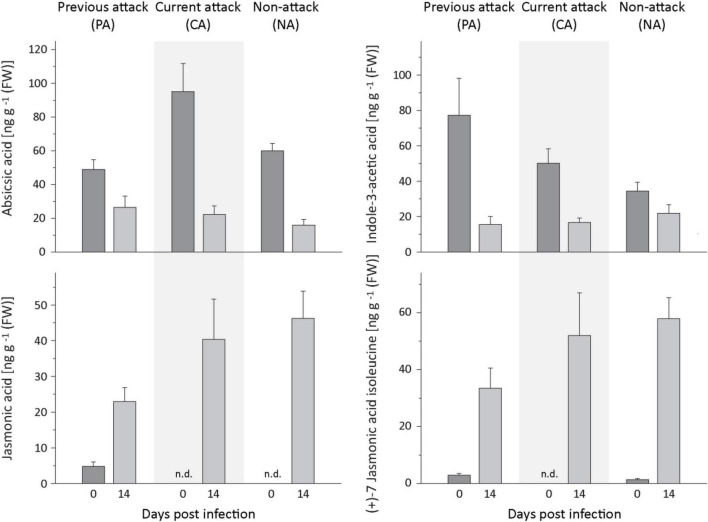
Quantification of phytohormones in spruce bark before and after *Endoconidiophora polonica* infection. The concentration of abscisic acid **(upper left panel)**, indole-3-acetic acid **(upper right panel)**, jasmonic acid **(lower left panel)**, and the (+)-7 jasmonic acid-isoleucine conjugate **(lower right panel)** were quantified by LC-MS/MS analysis from bark tissue harvested before and 14 days after infection with *E. polonica*. Trees investigated: (1) were from a stand with a history of previous bark beetle attack, but no current attack (PA), (2) were under current attack, but had no previous history of attack (CA), or (3) had no previous nor current attack (NA). Data are means ± SD of measurements from 6 to 12 biological replicates; exact numbers are given in the Section “Materials and Methods.” Statistical values for individual compounds are given in Supplementary Table 9.

The levels of several defense-related phytohormones in *P. abies* bark tissue were quantified to gain a better understanding of the signaling pathways involved in the defense response to *E. polonica* infection. The levels of abscisic acid (ABA) and indole-3-acetic acid (IAA) decreased significantly after inoculation with *E. polonica* (Figure 8 and Supplementary Table 9). Salicylic acid (SA) was not detected before or after inoculation of spruce bark. On the other hand, the concentration of jasmonic acid and the active (+)-7 jasmonic acid isoleucine conjugate increased significantly 14 days after induction after *E. polonica* inoculation (Figure 8 and Supplementary Table 9). For the jasmonic acid pathway, five additional metabolites were quantified (Supplementary Figure 4). The concentration of the jasmonic acid precursor 12-oxophytodienoic acid was significantly reduced after *E. polonica* inoculation but concentrations of the jasmonic acid degradation product, 12-hydroxyjasmonic acid, were not significantly altered after fungal inoculation (Supplementary Figure 5). However, the (−)-7 jasmonic acid isoleucine conjugate, considered to be a catabolite of the active (+)-7-conjugate, increased significantly after inoculation, while two degradation products of the active (+)-7-conjugate, the 12-hydroxyjasmonic acid isoleucine and the 12-carboxyjasmonic acid isoleucine conjugates, were only detectable after *E. polonica* inoculation (Supplementary Figure 4 and Supplementary Table 9). Due to the fact that not all phytohormones, their precursors and metabolites were detectable before fungal inoculation, differences between trees of different attack history prior to infection were not statistically evaluated.

## Discussion

### Bark Beetle Attack History Did Not Affect the Accumulation of Chemical Defenses After Fungal Infection

Inoculation of mature Norway spruce trees with the bark beetle-associated ascomycete *E. polonica* induced the jasmonate signaling pathway, leading to the increased accumulation of terpenes and phenolic compounds. The induction of chemical defenses in this conifer has been previously reported upon bark beetle attack and after wounding, methyl jasmonate treatment or inoculation with bark beetle fungal associates (Franceschi et al., 2002; Martin et al., 2002; Erbilgin et al., 2006; Heijari et al., 2008; Schmidt et al., 2011).

The extent of induction in this study was not dependent on bark beetle attack history. Trees that had been previously attacked did not show a greater degree of induction from those under current attack or those that had never been attacked. Thus, there was no priming effect of prior attack on the accumulation of terpene or phenolic defenses after the fungal inoculation we performed. Prior studies on Norway spruce have detected some evidence for defense priming, although the interval between the initial attack and subsequent challenge was much less than the 4 years in this study. For example, mechanical wounding inflicted one month after methyl jasmonate treatment gave an over 20-fold induction in terpenoids (Zhao et al., 2011). Curiously, in another study bark beetle attack 35 days after methyl jasmonate treatment did not lead to any significant increase in terpene content, yet trees were much more resistant to the beetles (Mageroy et al., 2020). Additional chemical and morphological defenses need to be screened to determine the basis of this resistance.

### Trees From Previously Attacked Stands Had Higher Levels of Some Terpenes and Phenolics

Independent of priming, trees that had been previously attacked by bark beetles may have had greater defense levels upon our fungal inoculation trial due to prior induction. Once formed, resin ducts persist in conifers for many years, while the phenolic-rich cells of the phloem remain intact for several years after formation and defend the tree (Nagy et al., 2000). In the present study trees with different bark beetle attack histories did not show significant differences in chemical defenses in most cases prior to our fungal inoculation. However, for the major monoterpenes, diterpenes and some phenolic compounds, previously attacked trees had higher levels than at least one of the other two categories (currently attacked or non-attacked trees) or both of them. These higher levels of defenses could have been induced by the original bark beetle attack and may help explain the survival of these trees. On the other hand, “previously attacked” trees may simply be survivors of the original attack that had never themselves suffered beetle damage. Constitutively high defense levels may have ensured their survival compared to less defended neighbors. We did not note any observable signs of prior beetle attack at sampling, and no anatomical studies were conducted.

### Induction of Terpene Biosynthesis and Accumulation Are Common Spruce Responses to Actual or Simulated Enemy Attack

After inoculation with the blue stain fungus *E. polonica*, the total terpene concentration in the bark of mature Norway spruce (*P. abies*) increased 3- to 9-fold with increases being generally larger for diterpenes (C_20_) and sesquiterpenes (C_15_) than monoterpenes (C_10_). Elevated terpene accumulation after *E. polonica* infection or methyl jasmonate (MJ) treatment (often employed as a surrogate for bark beetle attack) has been recorded in several previous studies (Viiri et al., 2001; Martin et al., 2002; Erbilgin et al., 2006; Zeneli et al., 2006; Zhao et al., 2010; Schmidt et al., 2011; Schiebe et al., 2012). However, the magnitudes of increase were variable with mature trees tending to exhibit greater accumulation than saplings. The induction of terpenoid in conifer stems is ascribed to the formation of additional resin ducts, known as traumatic ducts, in the developing xylem (Franceschi et al., 2002; Martin et al., 2002; Schmidt et al., 2010).

To determine if terpene accumulation after *E. polonica* fungal infection results from *de novo* biosynthesis as previously demonstrated after MJ treatment (Schmidt et al., 2010, 2011), the expression of biosynthetic genes was measured. Among the isoprenyl diphosphate synthases, the transcript levels of *IDS1*, encoding the formation of a joint GDP (C_10_) and GGDP (C_20_) synthase, *IDS4*, encoding the formation of an FDP (C_15_) synthase, and *IDS5*, encoding a GGDP (C_20_) synthase, were elevated on fungal infection in trees with all attack histories, except for *IDS1* and *IDS4* in previously attacked trees. In earlier studies on young Norway spruce saplings, *IDS1* and *IDS5* expression both increased after MJ application (Schmidt and Gershenzon, 2007; Schmidt et al., 2010), being elevated by more than 6-fold 22 days after treatment (Schmidt et al., 2011), whereas the expression of *IDS4* was not increased at all. Among the terpene synthase genes measured, the transcript level of monoterpene and diterpene synthases increased even more dramatically than those of the isoprenyl diphosphate synthases, but this was not true for the sesquiterpene synthases. Monoterpenes and diterpenes typically make up the bulk of conifer resin, with sesquiterpenes present in only minor amounts.

Along with the increase in *IDS* gene expression, there was an elevation in IDS enzyme activity producing GDP, FDP and GGDP and the *in vivo* concentration of GDP and FDP, but not GGDP, significantly increased, all consistent with a rise in terpene biosynthetic rate. After fungal inoculation, there was also a significant increase in the concentration of CDP, an intermediate in the conversion of GGDP to diterpene olefins by various conifer diterpene synthases (Peters et al., 2001). This increase paralleled the increases measured in diterpene synthase gene expression and terpene accumulation. Although *in vitro* studies with a recombinant conifer abietadiene synthase showed CDP to be present at a maximum of 2% of GGDP, we found CDP *in vivo* at levels of almost 20% of the GGDP concentration. This suggests that bifunctional diterpene synthases in *P. abies* (Martin et al., 2004) are not efficient in channeling CDP from the active site of the first reaction (conversion of GGDP to normal-CDP) to the site of the second reaction (conversion of normal-CDP to various diterpene olefins). The only diterpene synthases that are not bifunctional in spruce are *ent*-copalyl diphosphate synthase and *ent*-kaurene synthase, which are involved in gibberellin biosynthesis (Keeling et al., 2009). In contrast to *Taxus brevifolia* or *Ricinus communis* no diterpene synthases in spruce are known that use GGDP as a substrate and directly produce the diterpene product without the involvement of CDP (Zi et al., 2014).

In this study, prior bark beetle attack was not shown to prime the induction of terpene accumulation following fungal infection. Yet trees that had been previously attacked showed significantly higher expression of *IDS* genes involved in the formation of monoterpene and diterpenes than non-attacked trees or trees under current attack. Whether elevated transcript expression could be responsible for the priming of terpene formation under other conditions remains to be determined. It would be especially interesting to investigate the consequences of a genuine bark beetle attack, rather than artificial inoculation with a bark beetle-associated fungus.

### Induction of Phenolic Compounds by Fungal Infection May Be Influenced by Fungal Catabolism

Phenolic compounds also accumulated upon fungal infection in this study, but the increase was not as pronounced as for terpenes. Moreover, there was no effect of bark beetle attack history on the magnitude of accumulation. The increase in total phenolics was due to an increase of ∼2-fold in flavan-3-ol content, but no significant changes were detected in the levels of other flavonoids and stilbenes upon fungal infection, in agreement with earlier studies on fungus-infected *P. abies* saplings and mature trees (Viiri et al., 2001; Lieutier et al., 2003; Hammerbacher et al., 2014). Phenolics have also been shown to increase after MJ treatment (Erbilgin et al., 2006; Zeneli et al., 2006), but the pattern of accumulation is different compared to fungal infection. MJ applied to mature trees induced significant increases in all classes of phenolics measured (Schiebe et al., 2012). The lack of a broad increase after fungal infection may be ascribed to the ability of *E. polonica* to degrade certain host tree phenolics during infection (Hammerbacher et al., 2013; Wadke et al., 2016). The 3- to 5-fold increase of chalcone synthase transcript levels after fungal infection indicates that flavonoid biosynthesis was significantly enhanced by infection. Hence the lack of a measurable increase in flavonoids other than flavan-3-ols could well have resulted from fungal catabolism.

### Induction of Jasmonate but Not Salicylic Acid by *Endoconidiophora polonica* Is Consistent With Its Necrotrophic Lifestyle

The patterns of hormone signaling in response to fungal pathogens have long been known to depend on the lifestyle of the fungus. The response to necrotrophic pathogens typically involves both jasmonate and ethylene signaling, while the response to biotrophic and hemi-biotrophic pathogens is usually mediated by salicylic acid signaling (Glazebrook, 2005). In this study, infection of the bark of mature spruce trees with *E. polonica* induced the jasmonate pathway, but not the salicylic acid pathway, corresponding to the necrotrophic lifestyle of this fungus (Wadke et al., 2016). Jasmonic acid and several other jasmonic acid metabolites increased many-fold after *E. polonica* inoculation in all categories of tree samples regardless of their bark beetle attack history. However, no salicylic acid was detected either before or after inoculation in contrast to an earlier study involving the MJ treatment of 15-year-old trees, which used similar analytical methods (Schmidt et al., 2011). Both salicylic acid and jasmonic acid were present in significantly lower amounts than in woody angiosperms, such as in a poplar species attacked by insect herbivores (Clavijo McCormick et al., 2014), but the concentration of the active jasmonic acid isoleucine conjugate was comparable. Whether this difference reflects a true difference in jasmonate signaling between gymnosperms and angiosperms requires further study. In contrast to the jasmonates, the amount of abscisic acid declined significantly after fungal induction regardless of attack history. Unfortunately, our knowledge about the link between abscisic acid and defense in woody plants is still very limited. In poplar, abscisic acid was reported be induced by infection with a biotrophic fungus (Ullah et al., 2019).

## Conclusion

Infection of Norway spruce by the bark beetle-associated *E. polonica* resulted in the induction of a complex mixture of monoterpenes, sesquiterpenes, diterpene acids and flavan-3-ols in bark formed largely by *de novo* biosynthesis. These substances are believed to represent principal conifer defenses against the fungus and its bark beetle vector, serving as toxins, repellents, or physical barriers to invasion (Franceschi et al., 2005; Hammerbacher et al., 2011, 2013). No evidence was found that previous bark beetle attack primes the extent of defense induction although the 4-year-period between initial attack and our experimental fungal inoculation may have been too long for the maintenance of the priming signal. Prior to our induction, many of the terpene and phenolic defenses measured were higher in previously attacked trees than unattacked trees or those under current attack, likely a remnant of previous induction. Further research is required to understand the role of constitutive versus induced chemical defenses in protecting conifers against bark beetle invasion.

## Data Availability Statement

The original contributions presented in the study are included in the article/Supplementary Material, further inquiries can be directed to the corresponding author.

## Author Contributions

AH and AS designed the experiments and did the sampling. RN and AH performed the experiments and analyzed the data. GK performed the statistics. MP contributed to the molecular methods. JG and AS supervised the study. RN wrote the manuscript with contribution from AH, GK, MP, JG, and AS. All authors approved the submitted version.

## Conflict of Interest

The authors declare that the research was conducted in the absence of any commercial or financial relationships that could be construed as a potential conflict of interest.

## Publisher’s Note

All claims expressed in this article are solely those of the authors and do not necessarily represent those of their affiliated organizations, or those of the publisher, the editors and the reviewers. Any product that may be evaluated in this article, or claim that may be made by its manufacturer, is not guaranteed or endorsed by the publisher.
