# Supplementary material for: Bark Beetle Attack History Does Not Influence the Induction of Terpene and Phenolic Defenses in Mature Norway Spruce (Picea abies) Trees by the Bark Beetle-Associated Fungus Endoconidiophora polonica
Source: Front Plant Sci. 2022 May 6;13:892907. doi: 10.3389/fpls.2022.892907 (PMC9120863; doi:10.3389/fpls.2022.892907)
Supplement: Supplementary file 1 [file Data_Sheet_1.docx]

**Supplemental Figure 1**

**
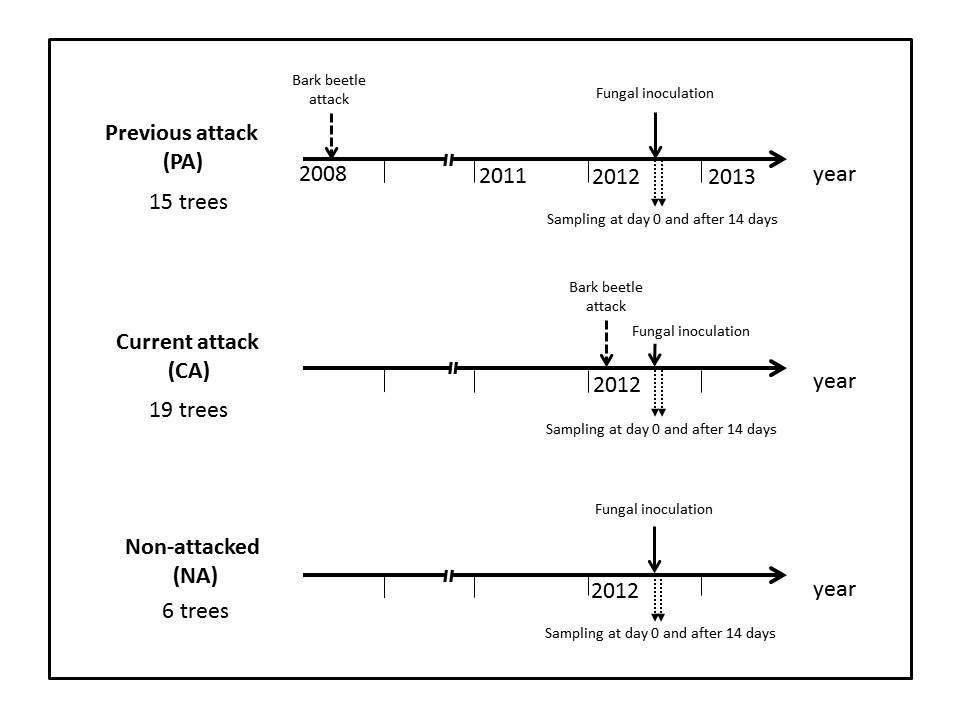
**

**Supplemental Figure 1 Illustration of the experimental design** A group of 15 mature spruce trees (*P. abies*), which were sole survivors of a massive bark beetle attack in 2008, was sampled in 2012, and designated as trees with a **p**revious **a**ttack history (PA). A group of 19 trees that were being attacked by bark beetles at the time of sampling was designated as trees under **c**urrent **a**ttack (CA). Six trees were sampled from a stand that was not affected by the current outbreak and had never been recorded to be attacked by bark beetles. These trees were designated as trees with **n**o current **a**ttack (NA). At the onset of the experiment, bark samples were cut from the stem and an 8-mm bark plug was removed on the opposite side of the stem pled stem and inoculated with *E. polonica* culture 1993-208/115 using the method of Hammerbacher et al. (2011). Bark samples were then taken from the inoculation site after 14 days.

**Supplemental Figure 2**


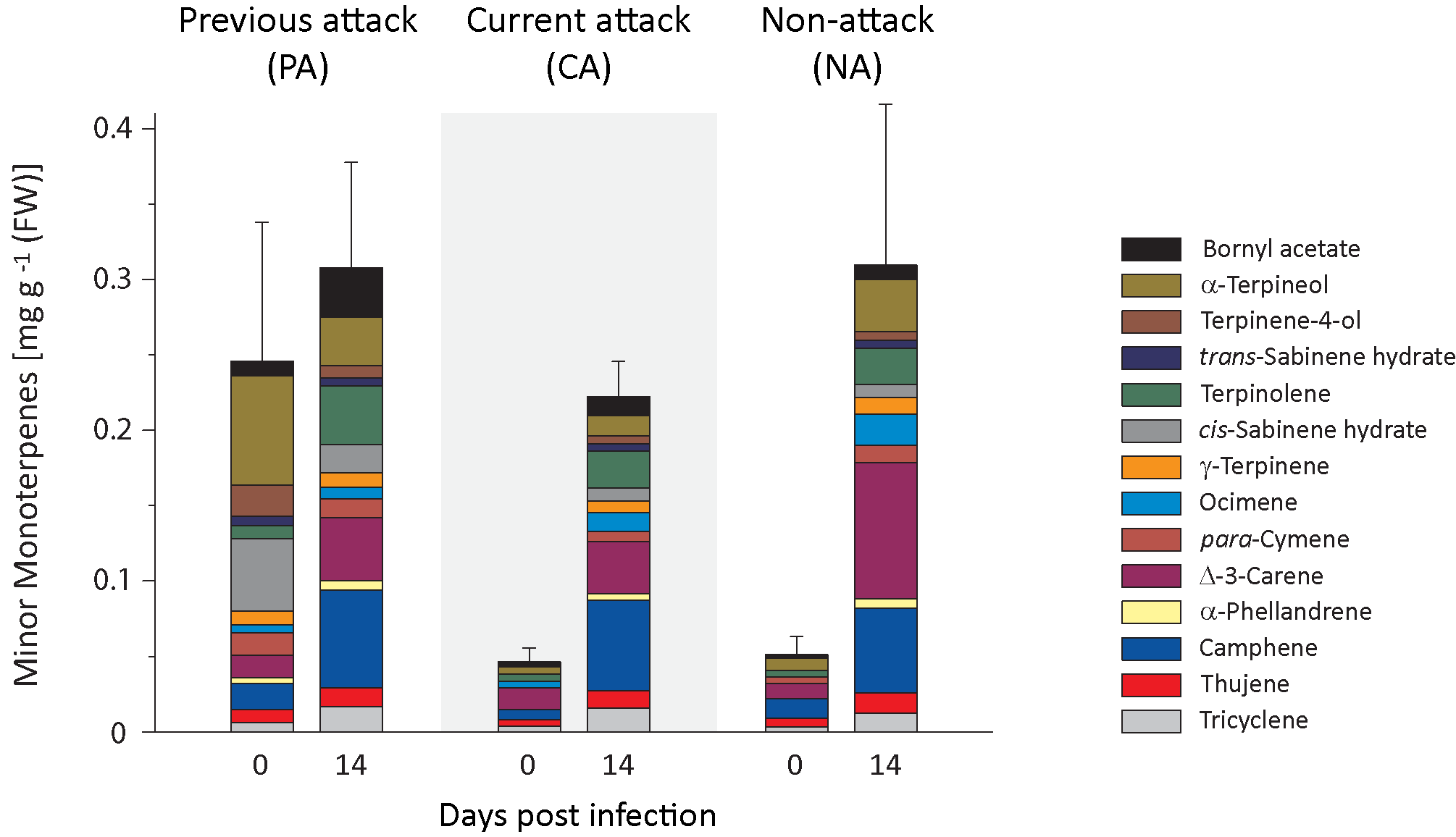


**Supplemental Figure 2: Quantification of minor resin monoterpenes in spruce bark before and after *Endoconidiophora polonica* infection.** The amounts were quantified by GC-FID from bark tissue harvested before and 14 days after infection with *E. polonica*. Trees investigated: (1) had a history of previous bark beetle attack, but no current attack (PA), (2) were under current attack, but had no previous history of attack (CA), or (3) had no previous nor current attack (NA). Data are means ± SD of measurements from 6–19 biological replicates, exact numbers are given in the Materials and methods section, Statistical values for individual compounds are given in Supplementary Table 2, 3.

**Supplemental Figure 3**


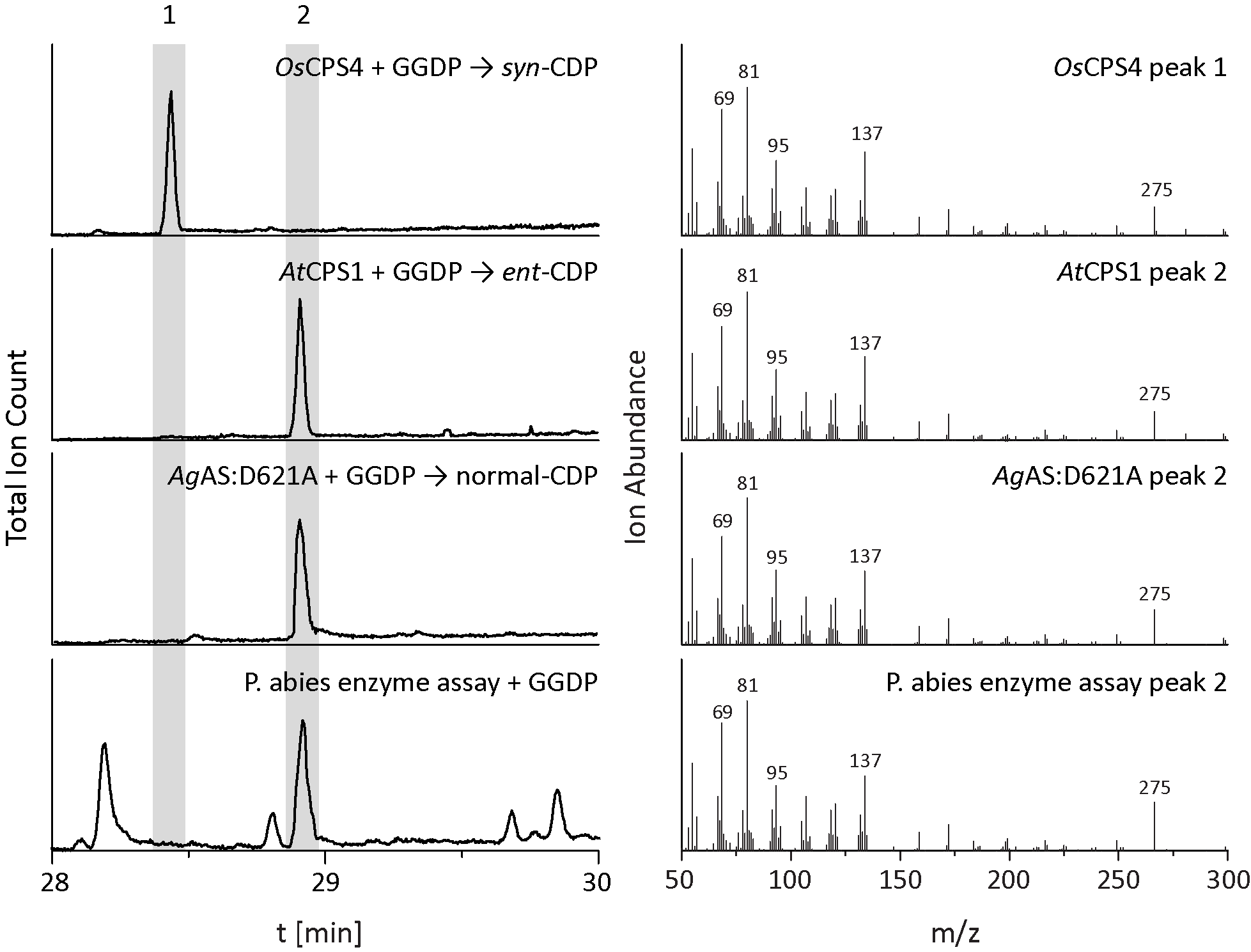


**Supplemental Figure 3: Determination of the stereochemical form of copalyl diphosphate (CDP) present in spruce bark.** Separation of the stereoisomers of copalyl diphosphate was not possible in the form of the diphosphate. However after enzymatic dephosphorylation the resulting alcohols could be separated by GC-MS into one peak for the *syn*- and one peak for the *ent-* and normal-stereoisomers. The left panel depicts the products of enzyme assay with *Os*CPS4 (makes *syn*-CDP) (Xu et al., 2004), *At*CPS1 (makes *ent*-CDP) (Prisic et al., 2004), *Ag*AS:D621A (makes normal-CDP) (Prisic et al., 2004) and *P. abies* total protein extract 14 days after inoculation (makes either *ent*- or *normal*-CDP). Right panel depicts the MS spectra of the indicated copalol peaks 1 or 2.

**Supplemental Figure 4**

**
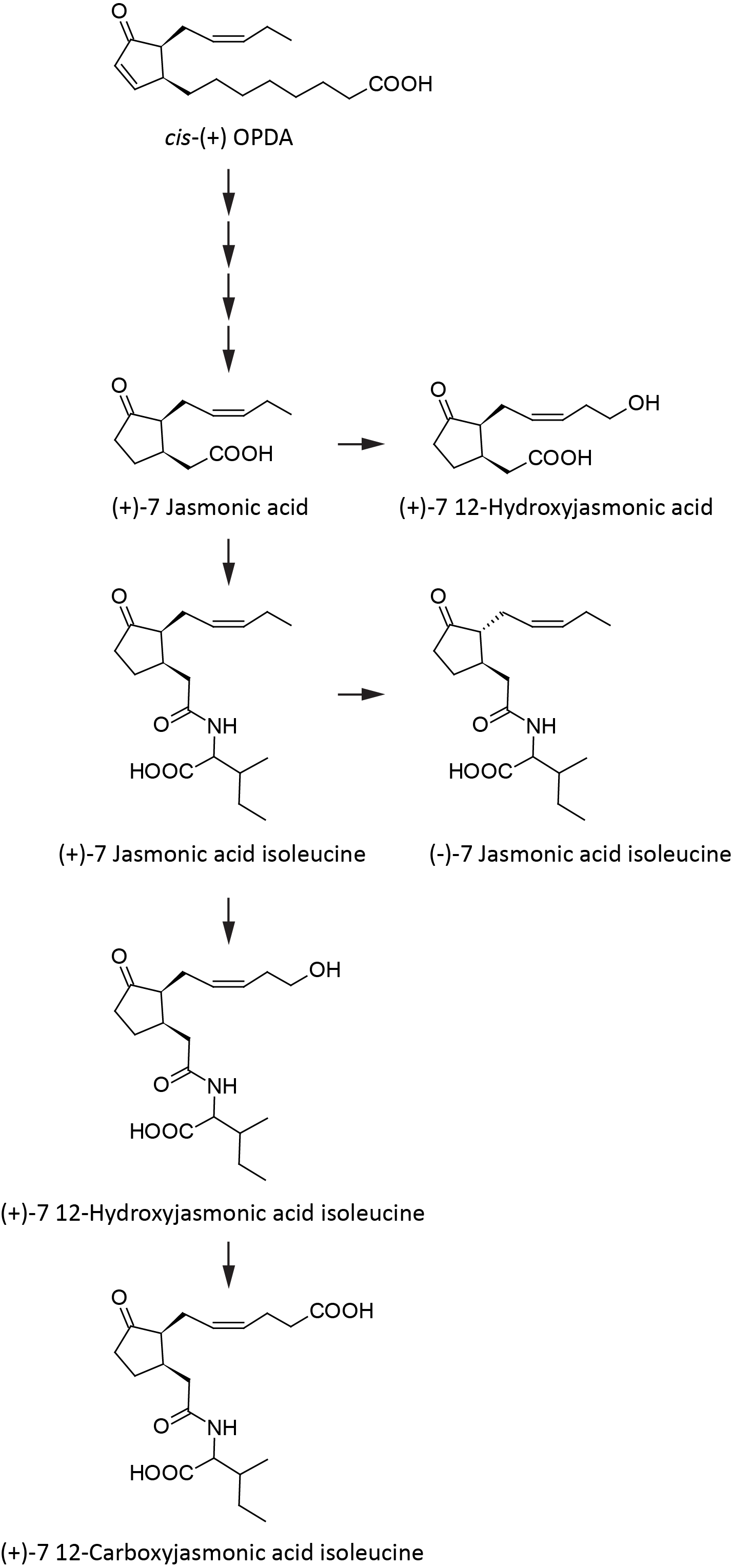
**

**Supplemental Figure 4: Outline of jasmonic acid biosynthesis and degradation showing metabolites that were measured in this study.** The (+)-7-jasmonic acid isoleucine conjugate is the bioactive form (modified from Wasternack and Hause, 2013).

**Supplemental Figure 5**


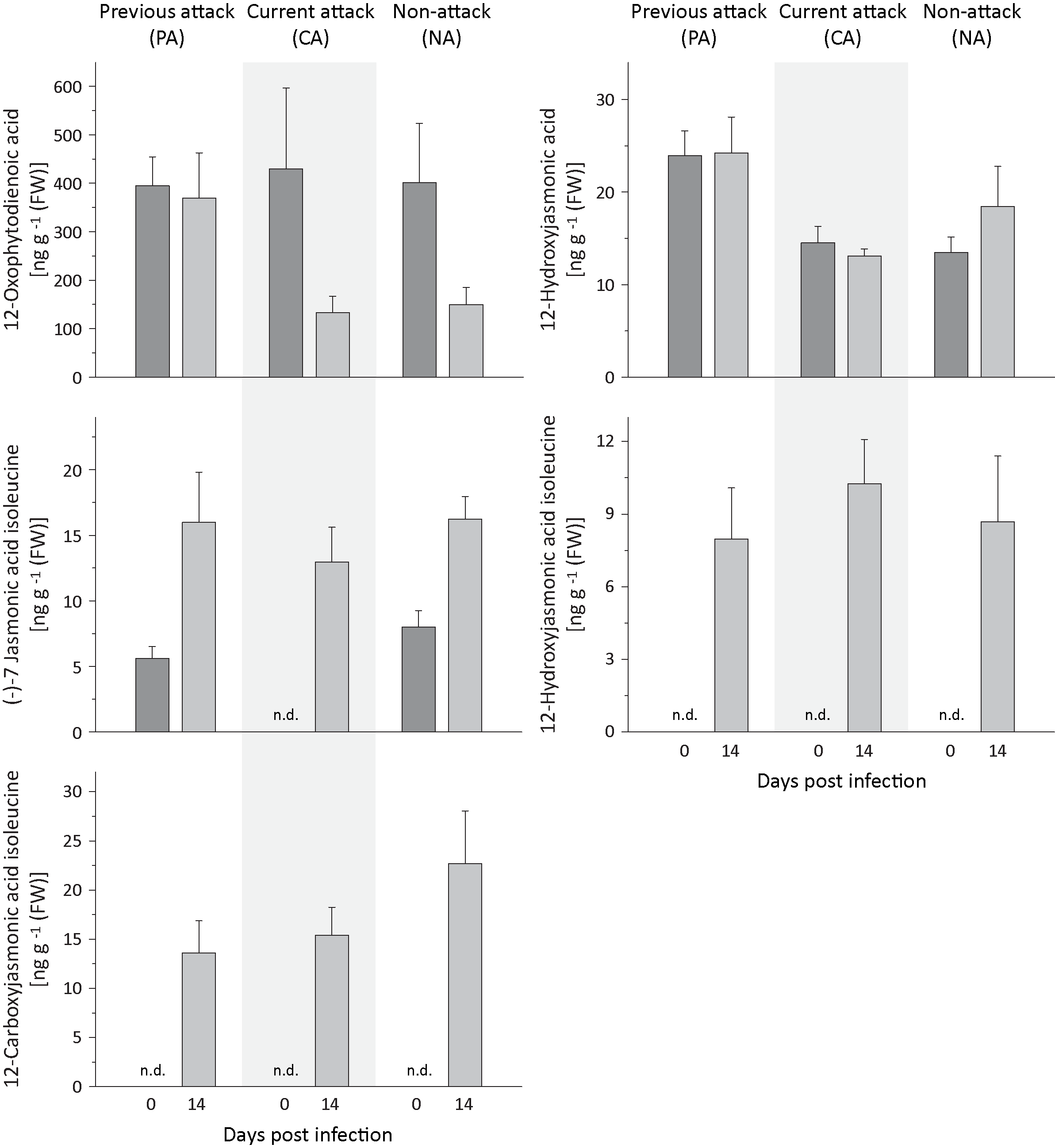


**Supplemental Figure 5: Quantification of jasmonic acid related metabolites in spruce bark before and after *Endoconidiophora polonica* infection.** The amount of 12-oxophytodienoic acid (upper left panel), 12-hydroxyjasmonic acid (upper right panel), the (-)-7 jasmonic acid isoleucine conjugate (middle left panel), the 12-hydroxyjasmonic acid isoleucine conjugate (middle right panel) and the 12-carboxyjasmonic acid isoleucine conjugate (lower panel) were quantified by LC-MS/MS from bark tissue harvested before and 14 days after infection with *E. polonica*. Trees investigated: (1) had a history of previous bark beetle attack, but no current attack (PA), (2) were under current attack, but had no previous history of attack (CA), or (3) had no previous nor current attack (NA). Data are means ± SD of measurements from 6–12 biological replicates, exact numbers are given in the Materials and methods section. Statistical significance for changes in individual compounds are given in Supplementary Table 9.

**Supplemental Table 1**

**Quantitative real-time PCR primers**

| Primer | 5' 🡪 3' |
| --- | --- |
| α-/β-Pinene Synthase Forward | GTT GTT GCT GAT CTG AAC TCA ACT GC |
| α-/β-Pinene Synthase Reverse | CGG GAA AGG CGA TGA GGG AA GC |
| δ-3-Carene Synthase Forward | GCA TCA CTT CTA CAA TTA TCG AGA TGG |
| δ-3-Carene Synthase Reverse | TGC AAC CTC AAA CTT TAG GCG |
| Bisabolene Synthase Forward | GCA GAC TGT TCT GGA TGA TAT GTA TG |
| Bisabolene Synthase Reverse | CTT CTC TAC CTC CCA AGC CAC |
| Longifolene Synthase Forward | TGT ACG CTT CCG ATA GAA ATT CTG C |
| Longifolene Synthase Reverse | GAC AAA ACG ATT CAT GGC TCA AGT CG |
| Diterpene Synthases Forward | GCC GTA CAG TGT TAT ATG AAG GAC C |
| Diterpene Synthases Reverse | CCA GTC TCC TGC AAC TGT CCG |
| Stilbene Synthases Forward | GTG GCG AGC AGA ACA CAG ACT TC |
| Stilbene Synthases Reverse | CAG CGA TGG TAC CTC CAT GAA CG |
| Chalcone Synthases Forward | CAG CAG TTC GGA ATC TCG GAC TGG AAC |
| Chalcone Synthases Reverse | CTC ATC TCG TCC AAG ATG AAG TGC ACG C |

**Supplemental Table 2**

**Statistical values for the analysis of changes in terpene concentrations after fungus inoculation of Norway spruce trees with different bark beetle attack histories.**

**Supplemental Table 3**

**Statistical values for the analysis of terpene concentrations of Norway spruce trees with different bark beetle attack histories before fungal treatment.**

**Supplemental Table 4**

**Statistical values for the analysis of phenolics concentrations after inoculation of Norway spruce trees with different bark beetle attack histories.**

**Supplemental Table 5**

**Statistical values for the analysis of phenolic concentrations of Norway spruce trees with different bark beetle attack histories before fungal treatment.**

**Supplemental Table 6**

**Statistical values for the relative expression of terpene and phenolic biosynthetic genes before and after inoculation of Norway spruce trees with different bark beetle attack histories.**

**Supplemental Table 7**

**Statistical values for the analyses of IDS enzyme activity after inoculation of Norway spruce trees with different bark beetle attack histories.**

**Supplemental Table 8**

**Statistical values for the analyses of prenyl diphosphate abundances after inoculation of Norway spruce trees with different bark beetle attack histories.**

**Supplemental Table 9**

**Statistical values for the analysis of changes in phytohormone concentrations after inoculation of Norway spruce trees with different bark beetle attack histories.**
